# Extended-spectrum β-Lactamase-producing Flora in Healthy Persons

**DOI:** 10.3201/eid1106.041111

**Published:** 2005-06

**Authors:** Camilla Rodrigues, Upasana Shukla, Simantini Jog, Ajita Mehta

**Affiliations:** * P.D. Hinduja National Hospital and Medicine Research Centre, Mumbai, India

**Keywords:** ESBL, Fecal carriage, Healthy Executives with no risk factors

**To the Editor:** Extended-spectrum β-lactamase (ESBL)–producing gram-negative bacilli are endemic in hospitals. In intensive care units, 2% prevalence of ESBL-producing organisms has been reported (1). Exceedingly high rates of ESBL-producing bacteria in Indian hospitals prompted us to look at the fecal carriage of ESBL in the community (2).

One hundred healthy executives received a comprehensive health check at our tertiary care center in central Mumbai from August to September 2004. The predominant isolates from stool samples obtained for routine examination were cultured, and initial screening for ESBL production was conducted by using the disk diffusion method according to NCCLS guidelines (3). For these isolates, the ESBL phenotypic confirmation was performed with ceftazidime-clavulanate for an increase in zone diameter by 5 mm (disk potentiation). In addition, the ATB BLSE strip (bioMérieux, Lyon, France) was used to confirm the presence of inhibitor (sulbactam)-susceptible enzymes and to differentiate the strains from those that were either inhibitor resistant or harboring other β-lactamases, such as those of AmpC derivation. The ATB BLSE strip consists of a varying concentration of ceftazidime, 0.5–32 mg/L, and aztreonam, 0.5–8 mg/L, with varying combinations of these agents with a β-lactamase inhibitor, i.e., + sulbactam, 0.06–1 mg/L. Cefotetan (4 and 32 mg/L) and imipenem (4 and 8 mg/L) were also included in the strip. The test was considered positive when a variation of ≥4 dilutions was observed between the antimicrobial agent tested alone and the agent combined with the inhibitor. Eleven of the 100 samples screened were positive for ESBL-producing *Escherichia coli* and *Klebsiella pneumoniae*. Seven of the 11 were confirmed by using the ATB BLSE strip. The MIC of ceftazidime and aztreonam in all 7 isolates was 8 μg/mL. We might be underreporting ESBL producers in these cases by not including the cefotaxime-clavulanate combination in addition to the ceftazidime-clavulanate concentration. The percentage resistance to ciprofloxacin was 45%. All isolates were susceptible to amikacin and the carbapenems. None of the executives gave a history of hospitalization in the last year or history of antimicrobial drug consumption in the last 6 months.

This trend in patients with no apparent risk factors for ESBL carriage calls for urgent attention. Unknown environmental factors are likely playing a key role in maintaining this selective pressure. Larger studies are required to substantiate these findings.
